# Active Monitoring of Fatigue Crack in the Weld Zone of Bogie Frames Using Ultrasonic Guided Waves

**DOI:** 10.3390/s19153372

**Published:** 2019-07-31

**Authors:** Jiajia Yan, Hashen Jin, Hu Sun, Xinlin Qing

**Affiliations:** School of Aerospace Engineering, Xiamen University, Xiamen 361005, China

**Keywords:** structural health monitoring, fatigue crack, sensor network, guided wave, bogie frame

## Abstract

The bogie frame is an important structure of railway vehicles, transmitting the traction, braking force, lateral force, and vertical force during the traction operation. With the development of high speeds and heavy loads, the appearance of fatigue cracks in the bogie frames is increasing, which reduces the driving life of railway vehicles and even causes serious traffic accidents. Real-time monitoring on the integrity of the bogie is an inevitable requirement for ensuring the safe operation of railway vehicles. In this paper, ultrasonic guided wave-based active structural health monitoring (SHM) was developed to identify the fatigue crack of the bogie frame. Experiments were conducted on a welded T-shape specimen with a thickness of 12 mm. A total of 10 piezoelectric lead zirconate titanate (PZT) disks were mounted around the weld zone of the specimen, five of which were used as actuators, and the other five were used as sensors. Five-peak modulation narrow-band sine waves were input into the actuators to excite the specimen. From the sensor signals, the advanced damage index (DI) was calculated to identify the propagation of the crack. The experimental results demonstrate that crack damage as small as 2 mm in the weld zone of the bogie frame can be successfully detected. Some practical issues for implementing the SHM in real applications, such as crack quantification and environmental compensation, were also discussed.

## 1. Introduction

As one of the most advantageous tools in urban intercity public transportation, railway vehicles have been highly valued in urban construction. With the rapid development of the modern economy, railway vehicles are developing towards high-speed and heavy-duty in order to improve railway transport capacity. Extensive attention has been paid to the safety of railway vehicles at this time, as railway vehicles are more and more popular. However, during past decades, railway vehicles accidents occurred frequently around the world, including different types of causes, such as collision, derailment, circuit failure, and equipment failure [1,2,3].

The bogie frame is an important structure of railway vehicles, transmitting the traction, braking force, lateral force, and vertical force during the traction operation [4]. As a full-welded structure, the stress concentration, high residual stress distribution, and potential welding defects in the weld zone can make its strength much lower than that of the metal base material. With the development of high speeds and heavy loads, the appearance of fatigue cracks in the bogies is increasing, which reduces the driving life of railway vehicles and even causes serious traffic accidents. Therefore, real-time monitoring of the structural integrity and early detection of minor damage hidden in the structure are needed to ensure the safe operation of railway vehicles, reduce sudden disastrous traffic accidents caused by structural failure, reduce maintenance costs, and extend service life.

Numerous nondestructive methods of inspecting railway vehicles have been investigated in the past. These methods range from simple visual inspection techniques to the most advanced non-destructive-testing (NDT) techniques. The NDT techniques include the ultrasonic technique, eddy current, and X-Ray [5,6,7,8,9], all of which need the bogie to be disassembled in order to perform the inspection. Structural health monitoring (SHM) is an important method to determine structural integrity. It involves the use of multidisciplinary areas, including structure, sensors, signal processing and interpretation, system integration, and computer science [10,11,12]. The objective of SHM technology is not only to detect structural damage, but also to provide early signs of physical damage. Many researchers have demonstrated that some active SHM methods can be used to effectively monitor fatigue cracks, such as ultrasonic guided wave (GW), intelligent coating monitoring (ICM) sensors, comparative vacuum monitoring (CVM) sensors, and flexible eddy current sensors [13,14,15,16]. Among these active SHM methods, ultrasonic guided wave-based active SHM technology using a built-in piezoelectric sensor network is being widely investigated and developed for detecting cracks in various types of metallic structures. Much research has been conducted on monitoring fatigue crack growth in metallic structures [17,18,19,20,21,22,23,24,25,26,27,28,29,30,31,32,33,34,35,36,37], including the physics-based damage index, relating sensor measurements to crack growth size [17], the interaction of Lamb wave modes at varying frequencies with a through-thickness crack of different lengths [20], Lamb wave mode decomposition using concentric ring and circular piezoelectric transducers [21], a multi-feature integration method based on a second-order multivariate regression analysis for the prediction of fatigue crack lengths using sensor measurements [24], and a Gaussian mixture model for monitoring crack propagation mixed in the time-varying influence [36].

In this paper, ultrasonic guided wave-based active SHM technology using a built-in piezoelectric sensor network is developed to perform rapid non-destructive evaluations in real time and provide long-term monitoring of the fatigue cracks in the weld zone of bogie frames. The active SHM system combines a built-in sensor network, portable diagnostic hardware, and data analysis software. Experiments are conducted on a welded T-shape specimen to verify the detectability of the developed SHM system.

## 2. Description of System Principles

**Figure 1:**
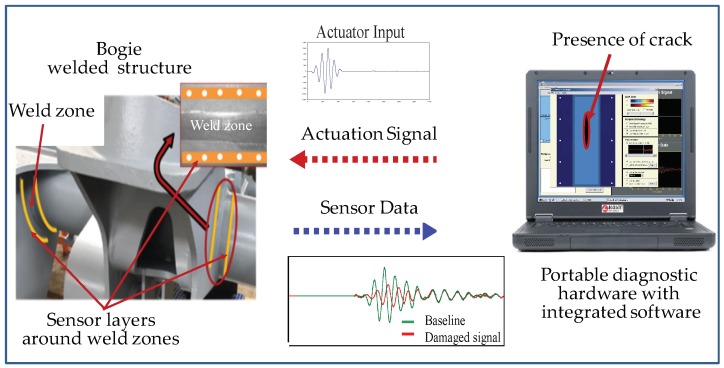
Schematic of an ultrasonic guided wave-based active structural health monitoring (SHM) system.

A schematic of an ultrasonic guided wave-based active SHM system for the monitoring of fatigue cracks in the weld zone of bogie frames is shown in Figure 1. The SHM system consists of a sensor network (sensor layers) permanently mounted around the weld zones being monitored, active diagnostic hardware for generating actuation signals and collecting sensor signals, and intelligent software for system control and signal processing. The sensor network of piezoelectric ceramic discs is often supported on a thin flexible dielectric film. The sensor layer offers a simple and efficient way to integrate the sensor network onto a structure. The piezoelectric ceramic discs on the layers can be used as either actuators or sensors. Once mounted, the sensor network can be used to collect health monitoring data throughout the service life of the structure. The diagnostic hardware consists of a signal generating unit, data acquisition unit, signal switching unit, data transmission cable, and storage device.

Five-peak modulated sine waves generated by the diagnostic hardware drive the actuators on the sensor layers to produce stress waves that propagate across the structure. The stress waves are measured by the sensors on the sensor layers. The sensor signals change when a crack occurs in the structure, since the propagating stress wave will be reflected or scattered by the crack. The diagnostic principle is based on the difference between the current sensor signals and previously recorded baseline sensor signals from the undamaged structure. The intelligent software uses the difference in these signals to identify the location and extent of crack. By using different signal processing techniques to process the diagnostic signals, information concerning the structure can be obtained for a variety of purposes.

## 3. Experimental Procedure

### 3.1. Design of Specimen

**Figure 2:**
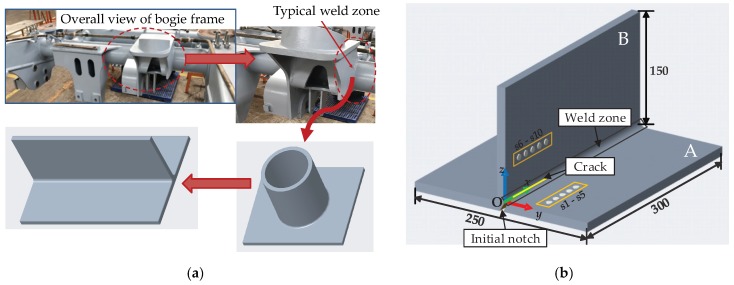
Design of specimen. (**a**) Simplification of bogie frame; (**b**) specimen with piezoelectric lead zirconate titanate (PZT) sensors (unit: mm).

According to the results of modal analysis and strength analysis, combined with the structure and technological characteristics of bogie frame, the weak parts of the bogie frame which are prone to fatigue damage during vehicle running mainly include four connection areas. They are the connection area of the frame side beam and rotating arm positioning beam base, connection area of the side beam and cross beam, connection area of the cross beam stop and brake hanger, and the connection area of brake hanger and cross beam [29]. There are two typical types of connection between the side beam of the welded box structure and cross beam of the seamless steel tube structure. One is the steel tube cross beam inserted into a round hole directly machined on the box side beam and welded together, the other is the box side beam and the cross beam connected by the transition seat. As shown in Figure 2a, in view of the existing typical connection forms, in order to facilitate the structural analysis of the connection between the bogie cross beam and the side beam, the T-shaped welded plate specimen made of steel Q235 was selected to stand for the typical welding structure between the cross beam and the side beam. The geometries of the T-shaped specimen are shown in Figure 2b.

As shown in Figure 2b, two steel plates with dimensions 300 mm × 250 mm × 12 mm (side A) and 300 mm × 150 mm × 12 mm (side B) were welded into a T-shaped specimen by argon arc welding. Ten piezoelectric lead zirconate titanate (PZT) sensors, each with a diameter of 8 mm and thickness of 0.48 mm, were mounted on side A and side B of the specimen using Hysol EA 9394 adhesive. The material parameters of PZT are listed in Table 1. Five PZTs, named s1, s2, …, s5, mounted on side A were used as actuators, the other five PZTs, named s6, s7, …, s10, mounted on side B were used as sensors. The distance between the actuators/sensors and center line of the weld zone was 50 mm. The locations of all PZTs are listed in Table 2.

### 3.2. Experimental Setup

The ScanGenie system from Acellent Technologies, Inc. (Sunnyvale, CA, USA) was used to generate actuating signals and record sensing signals. Five-peak sine waves, modulated by a cosine with central frequency at 90 kHz, 120 kHz, 150 kHz, 180 kHz, 210 kHz, 240 kHz, 270 kHz, 300 kHz, and 400 kHz, and an amplitude of 60 V, were used as the actuation signal inputs to piezoelectric actuators to excite the specimen. The neighboring sensors recorded the stress waves propagating through the specimen.

**Figure 3:**
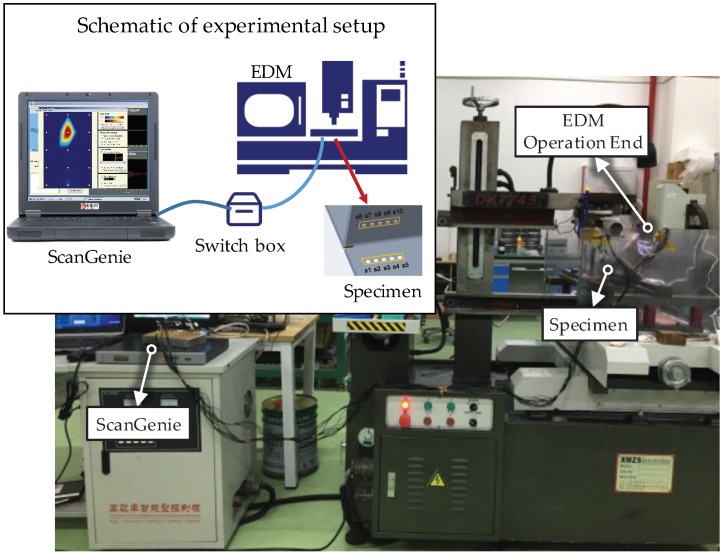
Experimental setup.

**Figure 4:**
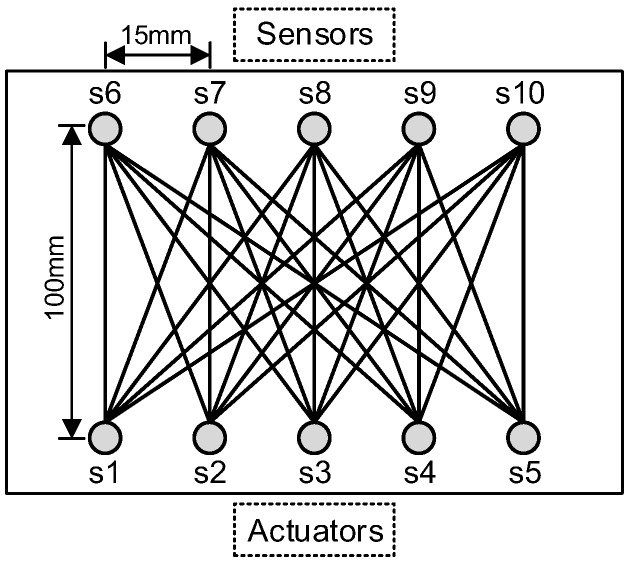
Actuator–sensor paths.

The experimental setup is shown in Figure 3. A notch, cut by an electrical discharge machining (EDM) device (Kingred DK-7740 from Huai′an Kingred CNC Technology Co., Ltd. (Suzhou, China), along the central line of the weld zone was used to simulate the fatigue crack. As shown in Figure 2, starting with the initial notch (to minimize the effect of the specimen edge), an EDM notch of 74 mm in length was introduced in the welding zone in 37 steps, increasing 2 mm with each step. Sensor signals for the actuator–sensor paths, such as s1–s6 (path 1–6), s2–s7 (path 2–7), s3–s8 (path 3–8), s4–s9 (path 4–9), and s5–s10 (path 5–10), shown in Figure 4, at different EDM notch lengths were recorded. It should be pointed out that the effect of the EDM notch on sensor signals was very similar to the effect of fatigue cracks when it is open, i.e., the difference between the EDM notch and fatigue cracks when opening does not affect the monitoring results. The effect of fatigue cracks when it is closed on the sensor signals is a little smaller than that when it is open.

### 3.3. Experimental Results

From the sensor measurements with exciting signals at different central frequencies, it was found that the sensor signals at frequencies below 200 kHz were very weak, and the change in sensor signals due to a small crack was not noticeable. At a higher frequency of 300 kHz, there were some complicated wave packets arriving at different times, which made the signal analysis very difficult. Therefore, the exciting signals with a central frequency of 240 kHz were used to generate stress waves in the specimen when the crack propagated.

**Figure 5:**
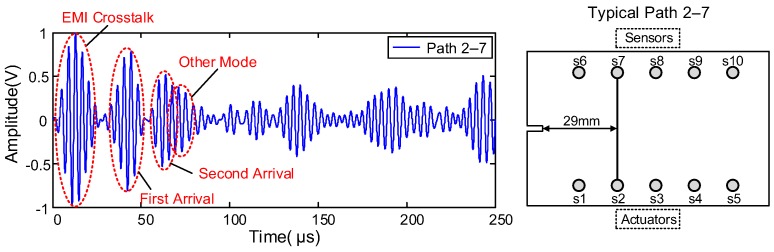
Baseline signal of path 2–7.

**Figure 6:**
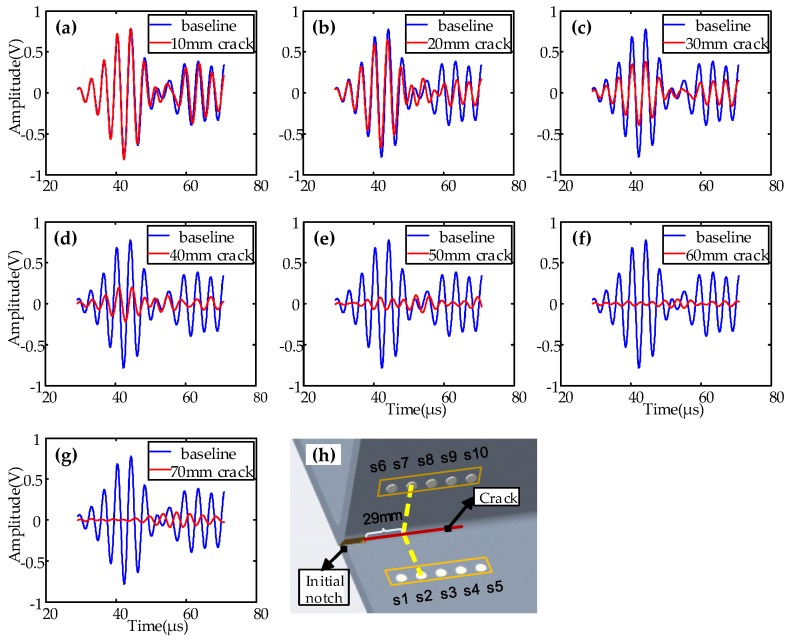
Comparisons of typical sensor signals of path 2–7 with different crack lengths (**a**) 10 mm crack; (**b**) 20 mm crack; (**c**) 30 mm crack; (**d**) 40 mm crack; (**e**) 50 mm crack; (**f**) 60 mm crack; (**g**) 70 mm crack; (**h**) schematic of specimen.

Typical signals with a central frequency of 240 kHz for path 2–7 are shown in Figure 5 and Figure 6. Figure 5 shows the baseline signal of path 2–7, which was 29 mm away from the initial notch. Figure 6 shows the sensor signals of path 2–7 at different EDM notch lengths (d = 10 mm, 20 mm, 30 mm, 40 mm, 50 mm, 60 mm, 70 mm). Similar sensor signals for other paths were obtained.

## 4. Analysis of Experimental Results

### 4.1. Monitoring Using Signals from a Single Actuator–Sensor Path

The TOF (time of flight), amplitude, and wave energy are three sorts of the most representative ultrasonic guided waves features which can be extracted from the captured signals [30]. The amplitude and wave energy are the most significant information characteristics in ultrasonic guided waves, which have proven effectiveness in locating gross damage [31]. The damage index (DI), calculated by various acoustic features of ultrasonic guided waves, is often used to characterize the length of crack. There are various methods to calculate DI, based on its definition in the time domain, frequency domain, and time–frequency domain [26]. In this paper, the following DIs calculated from the time domain were used to characterize the crack propagation in the welded zone of the T-shaped steel plate, and a comparative analysis was made among different DI definitions. The time windows used to calculate the DIs were determined based on the first arrival wave package.

1) DI based on amplitude:

(1) $$DI_{1}=\left|A_{1}-A_{2}\right|/A_{1};$$

2) DI based on root mean square deviation:

(2) $$DI_{2}=\sqrt{{\int \left[f_{2}\left(t\right)-f_{1}\left(t\right)\right]}^{2}dt/{\int \left[f_{1}\left(t\right)\right]}^{2}dt};$$

3) DI based on mean variance:

(3) $$DI_{3}=\left(1/T\right)\int {\left(f\left(t\right)-\bar{f}(t)\right)}^{2}dt;DI_{3}=\left(1/T\right)\int {\left(f\left(t\right)-\bar{f}(t)\right)}^{2}dt;$$

4) DI based on energy (Four Different Representations):

(4) $$DI_{4}=\left({\int \left|f_{1}(t)\right|}^{2}dt\right)/\left({\int \left|f_{2}(t)\right|}^{2}dt\right);$$

(5) $$DI_{5}=(\int {\left|f_{1}(t)-f_{2}(t)\right|}^{2}dt)/(\int {\left|f_{1}(t)\right|}^{2}dt);$$

(6) $$DI_{6}=\left|\int {\left[f_{2}(t)\right]}^{2}dt-\int {\left[f_{1}(t)\right]}^{2}dt\right|/\left(\int {\left[f_{1}(t)\right]}^{2}dt\right);$$

(7) $$DI_{7}=\left(\int {\left|f_{1}(t)\right|}^{2}dt-\int {\left|f_{2}(t)\right|}^{2}dt\right)/(\int {\left|f_{2}(t)\right|}^{2}dt);$$

5) DI based on amplitude attenuation:

(8) $$DI_{8}=\ln A_{2}/A_{1};$$

where *f*_1_(*t*) is the baseline signal, *f*_2_(*t*) is the current signal with crack, $A_{1}$is the amplitude of the baseline signal, and $A_{2}$ is the amplitude of current signal with crack propagation.

**Figure 7:**
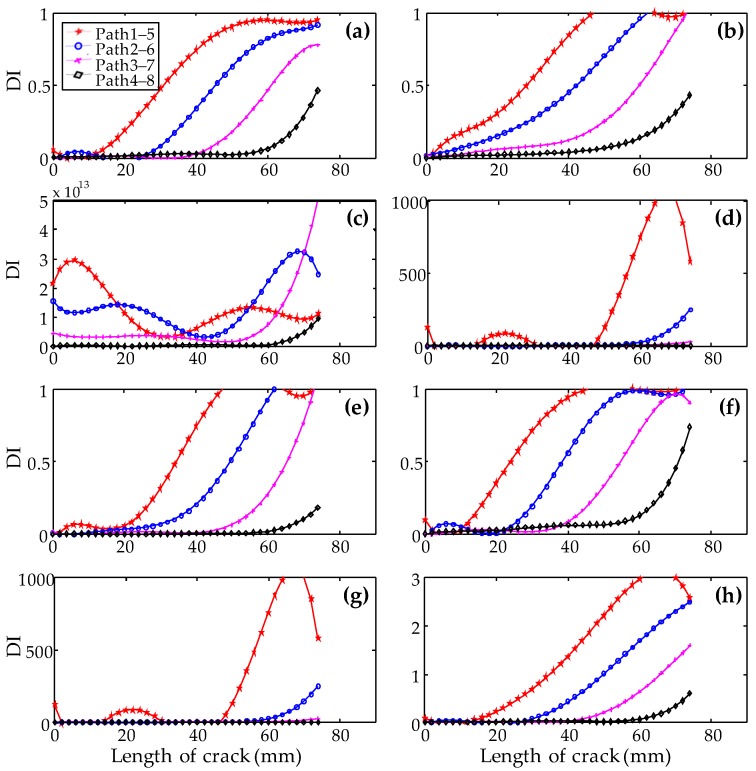
Damage index (DI) for typical paths calculated from different definitions (**a**) *DI*_1_ based on amplitude; (**b**) *DI*_2_ based on root mean square deviation; (**c**) *DI*_3_ based on mean variance; (**d**) *DI*_4_ based on energy; (**e**) *DI*_5_ based on energy; (**f**) *DI*_6_ based on energy; (**g**) *DI*_7_ based on energy; (**h**) *DI*_8_ based on amplitude attenuation.

Using the above different definitions, the DIs calculated from some typical paths are shown in Figure 7. It is obvious from Figure 7f that the DI curves based on energy definition Equation (6) show the clearest relationship with the lengths of cracks. The DI curves in Figure 7a,b,e,h from DI definition Equations (1), (2), (5), and (8) also show good relationships with the lengths of cracks. The DI curves in Figure 7c,d,g cannot be used to effectively identify the lengths of cracks. Therefore, the DI calculated from Equation (6) was used to monitor the propagation of cracks in the following investigation.

**Figure 8:**
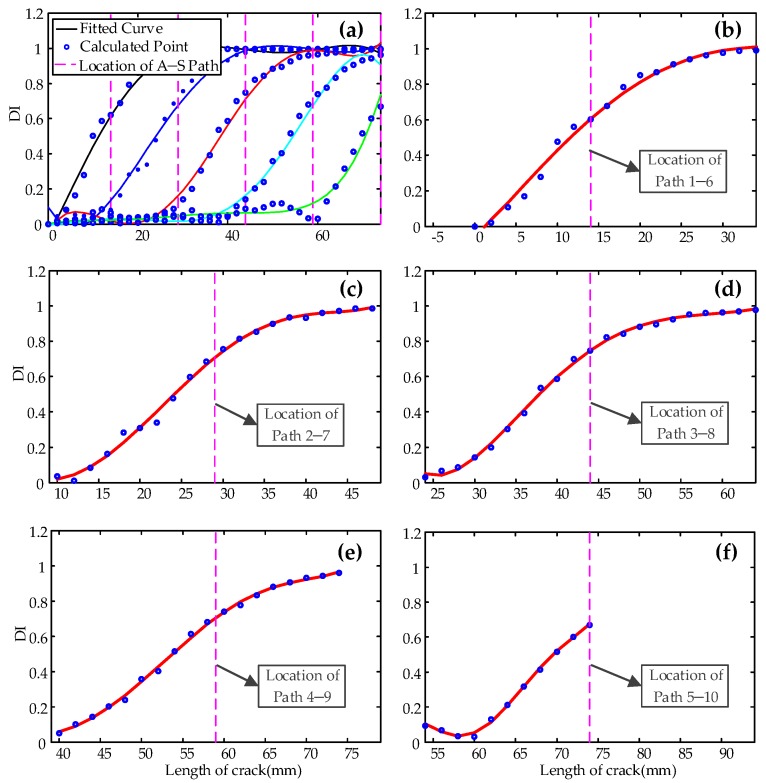
Damage index from (**a**) all the paths; (**b**) path 1–6; (**c**) path 2–7; (**d**) path 3–8; (**e**) path 4–9; (**f**) path 5–10.

The DIs calculated for typical paths 1–6, 2–7, 3–8, 4–9, and 5–10 independently at different crack lengths are shown in Figure 8. The dots in the figures denote the calculated DI value, and the solid curves are the fitted curves. In this paper, only the first arrival wave package of signal was used to calculate DI. Figure 8f only shows half of the DI curve for path 5–10 compared to other paths shown in Figure 8b–e, because the experiment was stopped when the crack length reached 74 mm.

**Figure 9:**
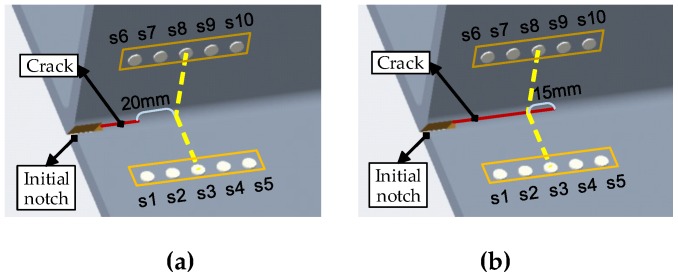
Affected area of the crack on the first arrival wave package of the signal. (**a**) Crack initially 20 mm from path 3–8 propagates to (**b**) 15 mm passed path 3–8.

It is clear from Figure 8 that the DIs calculated from all five paths change in the same trend. When the crack propagates toward the actuator–sensor path, the crack starts to affect the first arrival wave package of the sensor signal after the distance between the crack and the actuator–sensor path is less than 20 mm, as shown in Figure 9. When crack passes away 15 mm from the path, the amplitude of the first arrival wave package almost gets to zero. It is obvious that the DI from each path can be used to monitor the crack propagation in a bigger range when the second and more arrivals are used.

### 4.2. Monitoring Using Signal Fusion for All Actuator–Sensor Paths

It can be seen from Figure 8 that the DI from each path can be used to determine the propagation of the cracks in a certain range (about 35 mm) by using the first arrival wave package.

Aiming to make full use of the actuator–sensor paths to monitor the crack in the global area, a synthetic damage index related with fatigue crack was proposed to characterize the crack propagation covered by all paths. The synthetic DI can be defined as the following equation:

(9) $$DI=\frac{1}{5}\sum_{i=1}^{5}DI_{i}.$$

**Figure 10:**
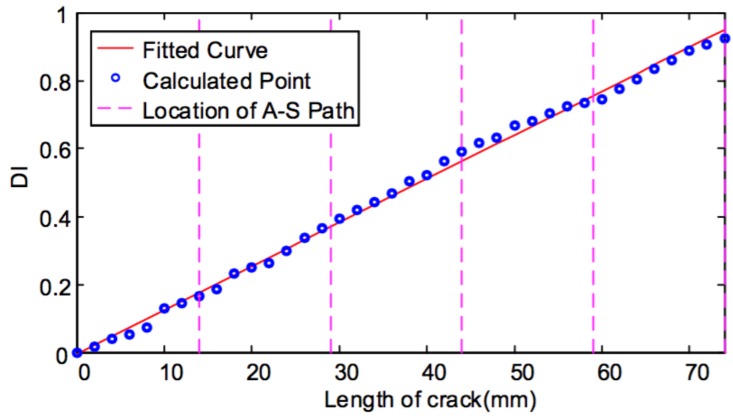
DI using signal fusion for all actuator–sensor paths.

When the crack in the weld zone of the specimen increases from the initial notch to 74 mm long, the synthetic DI calculated from the signal fusion for all actuator–sensor paths and its relationship with and length of crack is shown in Figure 10. It can be seen that as the crack propagates, the DI value increases approximately linearly.

## 5. Discussion

### 5.1. Ultrasonic Guided Waves in Steel Q235 Plate

**Figure 11:**
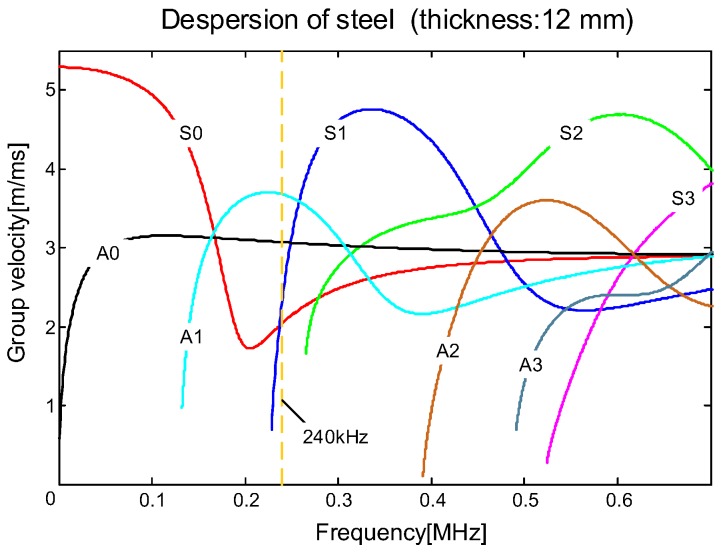
Dispersion curves of ultrasonic guided waves in a steel Q235 plate.

Figure 11 illustrates the dispersion curves of ultrasonic guided waves in a steel Q235 plate, calculated using DISPERSER^®^. The group velocity is the disseminated speed of the all wave packets. It can be seen that the A1 mode propagated at the fastest velocity among all available modes at the excitation frequency of 240 kHz, thus it can be completely separated from the other modes, boundary reflection, attenuation, and mode conversion signals.

**Figure 12:**
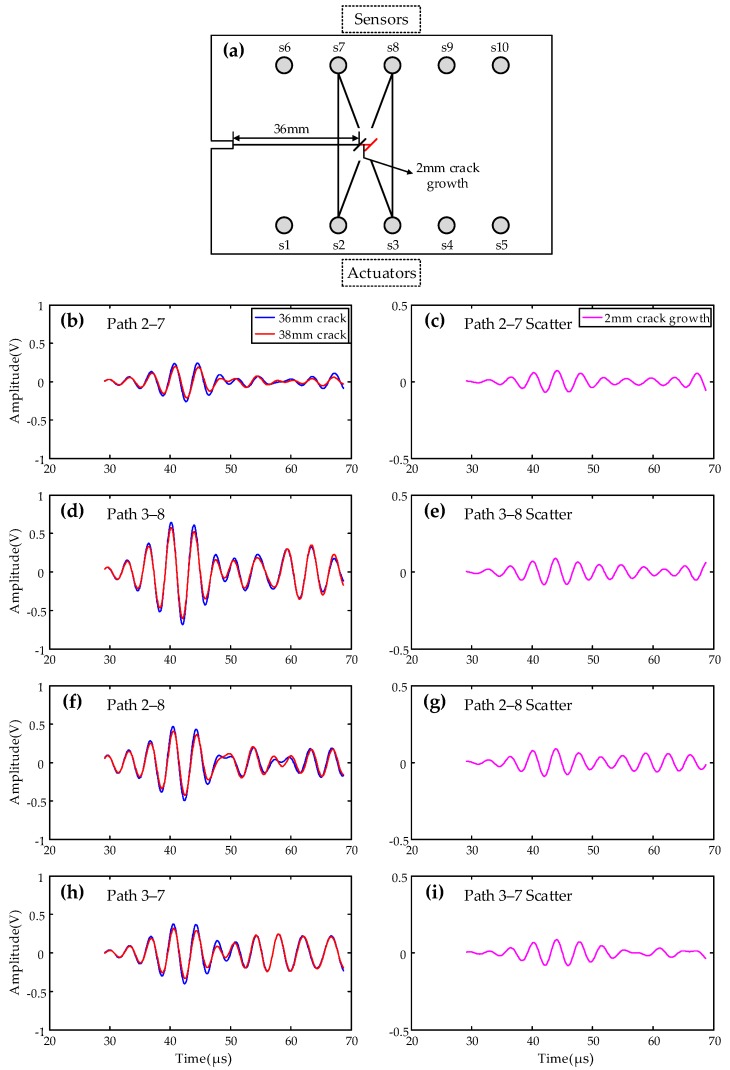
Comparisons of baseline and signal after 2 mm crack growth, diagnostic paths perpendicular or oblique intersection to the crack. (**a**) Schematic of actuator-sensor paths; (**b**) signals of path 2–7; (**c**) scatter of path 2–7; (**d**) signals of path 3–8; (**e**) scatter of path 3–8; (**f**) signals of path 2–8; (**g**) scatter of path 2–8; (**h**) signals of path 3–7; (**i**) scatter of path 3–7.

Taking the 24 mm crack as the baseline condition of the specimen, the comparisons of the baselines and signals of 2 mm crack propagation along actuation–sensor paths 1–6, 2–7, 1–7, and 2–6 are shown in Figure 12. It can be clearly seen that the signal changes are obvious due to the propagation of 2 mm cracks along these paths. This proves the ability of sensing technology to detect 2 mm cracks in the bogie.

### 5.2. Calibration Procedure for Crack Quantification

The DIs calculated from the change in sensor signals can be used to identify the propagation of the cracks. What was described above is the forward problem. According to the change in sensor signals resulting from the crack growth, the DIs were determined and the relationship between the DIs and crack length were obtained. However, in real applications, we need to solve the backward problem of how to accurately identify the crack size according the DIs. This is still a challenging task, even in a laboratory environment.

Based on the previous study [32], a semi-empirical method can be used to quantify fatigue crack growth in real time, giving few initial data points. DI curves are generated based on the signal changes from each path. To calibrate the DIs to the damage sizes, several measurements of real crack size measured by conventional methods are utilized. For instance, a few measurements of crack size can be made to determine the slope of the relationship curve between the DI and crack size, as shown in Figure 10. It is obvious that the more measurements are used, the better the estimates of the crack size will be.

In addition, the Lamb wave signal processing methods usually first extract the signal features, such as time of flight, amplitude, energy, and correlation coefficient, comparing with the reference signal to construct the DI. These methods assume that the environmental conditions stay unchanged. However, the railway vehicles usually work in a time-varying environment, by which the above features of the Lamb wave will be directly and significantly affected. It is difficult to obtain accurate damage information by using the damage detection methods without considering environmental factors. Therefore, environmental compensation methods need to be further investigated to ensure the implementation of developed active SHMs on monitoring the fatigue cracks of bogies in the real world.

## 6. Conclusions

Ultrasonic guided wave-based active SHM technology was investigated and developed for monitoring crack growth in the weld zone of bogie frames. The active SHM system consisted of a sensor network permanently mounted around the weld zone being monitored, diagnostic hardware for generating actuation signals and collecting sensor signals, and intelligent software for system control and signal processing. Experiments were conducted on a welded T-shape specimen with a thickness of 12 mm. The damage index was extracted from the Lamb wave signals to characterize the length of the crack. From the experimental results, it is clear that a 2 mm crack can be effectively detected if it is located in the range of about 20 mm toward the actuator–sensor path and 15 mm away the path when the first arrival wave package is used. Making full use of multiple actuator–sensor paths, the propagation of the crack in the weld zone of the specimen can be monitored in a big range, from the baseline condition to 74 mm long.

## Figures and Tables

**Table 1:** Material parameters of the PZT piezoelectric sensor.

Type	Density (kg\m^3^)	Curie Temp (°C)	Diameter (mm)	Thickness (mm)	Dielectric Constant	$d_{31}$	$d_{33}$
PZT-5A	7750	360	8	0.33	1700	-200	530

Note: *d*_31_, *d*_33_ are piezoelectric constants (×10^−12^ C/N).

**Table 2:** Locations of the PZT sensors (unit: mm).

	s1	s2	s3	s4	s5	s6	s7	s8	s9	s10
x	30	45	60	75	90	30	45	60	75	90
y	50	50	50	50	50	0	0	0	0	0
z	0	0	0	0	0	50	50	50	50	50

## Nomenclature

$d_{31}$, $d_{33}$	piezoelectric constants
*sn*	the number of sensors
$DI_{n}$	damage index
$A_{1}$	the amplitude of baseline signal
$A_{2}$	the amplitude of current signal
$f_{1}\left(t\right)$	the baseline signal
$f_{2}\left(t\right)$	the current signal
$\bar{f}(t)$	the average of signal
$S_{n}$(n = 0,1, …)	the nth symmetrical mode
$A_{n}$(n = 0,1, …)	the nth asymmetrical mode
